# Qualitative and quantitative analysis of self-reported sensory issues in individuals with neurodevelopmental disorders

**DOI:** 10.3389/fpsyt.2023.1077542

**Published:** 2023-02-09

**Authors:** Makoto Wada, Katsuya Hayashi, Kai Seino, Naomi Ishii, Taemi Nawa, Kengo Nishimaki

**Affiliations:** ^1^Developmental Disorders Section, Department of Rehabilitation for Brain Functions, Research Institute of National Rehabilitation Center for Persons With Disabilities, Tokorozawa, Japan; ^2^Information and Support Center for Persons With Developmental Disorders, National Rehabilitation Center for Persons With Disabilities, Tokorozawa, Japan; ^3^Psychological Experiment Section, Department of Social Rehabilitation, Research Institute of National Rehabilitation Center for Persons With Disabilities, Tokorozawa, Japan; ^4^Hospital of National Rehabilitation Center for Persons With Disabilities, Tokorozawa, Japan

**Keywords:** sensory issue, developmental disorder, quality of life, hypersensitivity, autism spectrum disorder

## Abstract

**Introduction:**

Individuals with neurodevelopmental disorders, such as autism spectrum disorder (ASD), attention-deficit/hyperactivity disorder (ADHD), and specific learning disorders (SLD) have various types of sensory characteristics.

**Methods:**

This study investigated sensory issues in individuals with neurodevelopmental disorders using a web-based questionnaire for qualitative and quantitative analysis, categorized the contents of their three most distressful sensory issues, and evaluated their order of priority.

**Results:**

Auditory problems were reported as the most distressing sensory issue among the participants. In addition to auditory problems, individuals with ASD frequently reported more tactile problems, and individuals with SLD reported more visual problems. Among the individual sensory issues, in addition to aversion to sudden, strong, or specific stimuli, some participants reported confusions regarding multiple stimuli presenting concurrently. Additionally, the sensory issues related to foods (i.e., taste) was relatively more common in the minor group.

**Conclusion:**

These results suggest that the diversity of sensory issues experienced should be carefully considered when aiding persons with neurodevelopmental disorders.

## 1. Introduction

Atypicality in social communication and restricted and repetitive behaviors or interests are major characteristics of individuals with autism spectrum disorder (ASD), as described in the Diagnostic and Statistical Manual of Mental Disorders, fifth edition (DSM-5), published by the American Psychiatric Association (APA) in 2013. Such conditions are accompanied with a wide variety of sensory characteristics, and the DSM-5 states that excessive or restricted responses to sensory stimuli—hypersensitivity and hyposensitivity, respectively—are often observed in individuals with ASD. Sensory issues in individuals with ASD, such as hypersensitivity and hyposensitivity, are not minor issues but are important factors pointing to the core of the disorder because some of these sensory characteristics directly decrease individuals’ quality of life [QoL; (1)]. Additionally, it is possible that differences in sensory processing between individuals with ASD and typically developing individuals may cause discrepancies in communication methods (2, 3).

A total of 60% to 90% of individuals with ASD experience sensory issues (4–10). Although difficult to generalize, individuals with ASD show a range of sensitivity preferences (including hypersensitivity and hyposensitivity), sensory distortions, overload, multichannel receptivity, and processing difficulties (11). Sensory issues in individuals with ASD are evident in auditory, visual, and tactile sensory modalities (12), and meta-analytic studies have indicated the existence of atypical sensory modulation in many individuals with ASD (13, 14).

Moreover, in addition to ASD, attention-deficit/hyperactivity disorder (ADHD) and specific learning disorder (SLD) are also included in neurodevelopmental disorders in the DSM-5, and these may also cause sensory issues, similar to ASD. For example, individuals with ADHD also show high scores for sensory sensitivity and sensation avoidance (15); although, it is difficult to determine how much of this is owing to a potential overlap with ASD. Neuroimaging studies have revealed shared alterations in the brain’s white matter and its connections with ASD regarding hypersensitivity (16, 17). Contrastingly, higher visual processing scores were observed in children with ADHD, compared to children with ASD and typically developing children, while oral processing scores were highest in children with ASD (18). These reports suggest that there are shared but partially distinct sensory features in between individuals with ASD and ADHD.

Among SLD, Irlen syndrome has been described as individuals having low reading ability owing to low color matching and distorted vision (19–21). Patients with this syndrome also experience visual hypersensitivity. Currently, the diagnoses of ASD, ADHD, and SLD may overlap (22), and the degree of overlap varies across individuals. We speculate that there are many differences in their sensory issues, and understanding these differences is important for the development of clinical support.

Auditory problems are major sensory issues in individuals with ASD (10, 23, 24). That is, such individuals feel distressed by some kind of sound or sometimes find it difficult to listen to someone talking. Problems related to touch, smell, and taste lead to serious difficulties, such as maladaptation to the living environment and selective eating problems (25, 26). Additionally, individuals with ASD are sometimes distressed by glaring lights (27). Contrastingly, some individuals with ASD tend to be insensitive to pain and temperature (28), and hypersensitivity and hyposensitivity are sometimes co-localized (12). However, owing to the great diversity of sensory issues, further investigation is needed to determine the sensory issues of individuals with neurodevelopmental disorders (13, 29).

Dunn and Westman (30) established a classification and evaluation method for sensory issues to provide intervention support. According to them, the two axes of threshold values for sensory stimuli and high activity of the participant were divided into independent quadrants of “low registration,” “sensory seeking,” “sensory sensitivity,” and “sensation avoidance.” A quadrant with a high threshold value for sensory stimuli and low activity was defined as “low registration,” whereas a quadrant with a high threshold value for sensory stimuli and high activity (i.e., seeking sensory stimuli) was classified as “sensory seeking.” Contrastingly, they defined a quadrant with a low threshold value for sensory stimuli and low activity as “sensory sensitivity” (hypersensitivity), and that with a low threshold value for sensory stimuli and active effort to avoid it as “sensation avoidance.” Subsequently, the adult/adolescent sensory profiles were standardized based on this classification (31).

Using the existing questionnaire (e.g., “Sensory Profile”) for each sensory issue, an appropriate support plan can be developed by evaluating the issues of each participant. Thus far, many previous studies that assessed sensory issues have used existing questionnaires [Sensory Profile, Sensory Sensitivity Questionnaire-Revised, Sensory Experiences Questionnaire, etc.; (13, 14)]. Several studies have attempted to classify the sensory features among individuals with ASD using scores from these questionnaires, and the existence of ASD subgroups widely documented (32–36). For example, some of these subgroups are characterized by difficulties with taste, smell, movement, and energy regulation (35, 36). Therefore, the existing questionnaires are very useful for such clustering and comparisons among groups.

Notably, since sensory characteristics of neurodevelopmental disorders seem to be diverse and have many aspects as reported previously (37), there are likely to be issues that are not covered by existing questionnaires. Therefore, we used a free-writing field for sensory issues as well as multiple-choice questions to clarify and categorize diverse sensory issues. This may be important for providing evidence-based support for individuals experiencing sensory issues. Moreover, it is important from a support perspective to evaluate a priority among these sensory issues. Hence, in this study, we investigated sensory issues in individuals with neurodevelopmental disorders using a web-based questionnaire for qualitative and quantitative analysis and examined a hierarchy of the most distressful sensory issues for each person.

## 2. Materials and methods

### 2.1. Questionnaire

We targeted participants who had a diagnosis (or suspicion) of neurodevelopmental disorders (ASD, ADHD, SLD, intellectual disability, and others), which are defined as “発達障害” (developmental disorders) in the Act on Support for Persons with Developmental Disorders in Japan. The original web-based questionnaire was written in Japanese (Supplementary material). First, an explanation of the study purpose was provided to participants, and after confirming the consent to participate in the study by ticking a check box, participants were asked to answer the questionnaire (in case of minors, their guardians needed to agree on their behalf). Multiple-choice questions and free-writing fields were combined in the questionnaire. Multiple-choice questions that identified the respondent’s gender, age, responder (concerned individuals themselves, support providers, or parents), current position regarding employment or educational status, and diagnosis were presented first. Subsequently, participants had to choose the modality of the most distressful sensory issues that they experienced (i.e., “visual,” “auditory,” “tactile,” “taste,” “olfactory,” “proprioception,” “vestibular,” and “others”). Thereafter, a free-writing field was provided to describe concrete examples of these issues. The free-writing field asked, “Please describe concrete examples of your issues. What are the triggers and reasons for their occurrence?” In case participants recognized second and third-most distressing issues; similar questions were presented to identify these distressing issues. For each sensory issue, questions about severity and restrictions in daily life using a Likert-type scale (0: *not severe* to 5: *most severe*, and “unanswerable”) were also presented (Supplementary Table S1). Additionally, the questionnaire included additional free-writing fields (about self-coping) and questions regarding where and when the problems arose; these were preliminary investigations for future studies (Supplementary material).

The questionnaire items were co-developed with the cooperation of volunteer group members (“OhToT”) who discussed new support methods for neurodevelopmental disorders. Individuals with ASD/ADHD/SLD, researchers, occupational therapists, engineers, medical doctors, and administrative officers joined this group. Thus, the persons who concerned to neurodevelopmental disorders were directly involved in this study.

### 2.2. Participants and procedure

The questionnaire was posted on the website of the Developmental Disorders Information and Support Center of the National Rehabilitation Center for Persons with Disabilities, Ministry of Health, Labor and Welfare of Japan. The response period was from August 2018 to January 2019, during which 432 responses were received. There was one duplicate response in which all items were completely matched to another response, which was removed from further analysis. Furthermore, because the contents of some descriptions included issues other than sensory issues (e.g., difficulties related to communication), we deleted these responses from further analysis (16 cases in the “most distressful” sensory issue category); thus, 415 responses were finally included. The demographic characteristics such as sex, age range, responder information, and current positions are shown in Table 1.

**Table 1:** Participants’ demographics.

A. Gender	
Male	172
Female	230
Not specified	13
Total	**415**
B. Age range (years)	
<6	7
<12	50
<18	56
<23	38
<30	65
<40	61
<50	84
<60	51
<70	3
Total	**415**
C. Responder	
In person	274
Parent	114
Supporter	27
Total	**415**
D. Current position	
Preschool	12
Elementary school	53
Junior high/high school	46
Special support schools	14
Higher education	25
Working	174
Working as a welfare worker	40
Other	79
Total (including duplicated responses)	443

For current position, 28 duplicated responses (14 participants) are included. Bolded numbers indicate total.

This research was reviewed and approved by the Ethics Review Committee of the National Rehabilitation Center for Persons with Disabilities (29–175, 30–154, 31–109, 2021–136) and was conducted in compliance with Declaration of Helsinki and the “Medical Research Guidelines for Humans” of the Ministry of Health, Labor and Welfare of Japan.

### 2.3. Analysis

Responses were analyzed using Microsoft Excel (Office2019, Microsoft, Redmond, WA, United States). Participants were asked to answer a multiple-choice question regarding their diagnoses; possible answers included, “ASD (including autism, Asperger’s syndrome, and pervasive developmental disorders),” “ADHD,” “SLD,” “intellectual disabilities,” “others,” and “none (including suspicion).” Additionally, there was a free-writing field to specify the situation for “others” and “none” responses below the multiple-choice question. If the response of the free-writing field included “suspicion of ASD/ADHD/SLD at clinic,” the person was included in the ASD/ADHD/SLD group, respectively. There was overlap in the diagnosis or suspicion; thus, to clarify the differences in the characteristics of sensory issues in ASD, ADHD, and SLD, three types of classification were performed as follows.

First, participants were divided into two groups: those with ASD (*n* = 281, ASD group) and those without ASD (*n* = 134, non-ASD group). The ASD group included 11 ASD-suspected cases (Table 2A). Second, participants were divided into two groups: those with ADHD (*n* = 164, ADHD group) and those without ADHD (*n* = 256, non-ADHD group). The ADHD group included five ADHD-suspected cases (Table 2B). Third, participants were divided into two groups: those with SLD (*n* = 56, SLD group) and those without SLD (*n* = 361, non-SLD group). The SLD group included two SLD-suspected cases (Table 2C). Then, cross-tabulation was performed between the groups (i.e., “ASD group” vs. “non-ASD group;” “ADHD group” vs. “non-ADHD group;” and “SLD group” vs. “non-SLD group”), and *Χ^2^* tests (test of independence) were performed to examine statistical differences between groups.

**Table 2:** Diagnosis of participants.

A. Diagnosis of ASD	
ASD	270
Suspicion	11
Non-ASD	134
Total	**415**
B. Diagnosis of ADHD	
ADHD	159
Suspicion	5
Non-ADHD	251
Total	**415**
C. Diagnosis of SLD	
SLD	54
Suspicion	2
Non-SLD	359
Total	**415**

Overlapping cases were included in above tables (ASD + ADHD: 76, ASD + SLD: 11, ASD + ADHD + SLD: 19, ADHD + SLD: 9, ASD suspicion + ADHD suspicion: 3). Bolded numbers indicate total.

For the free-writing field (“Please describe concrete examples of issue. What are the triggers and reasons for their occurrence?”), four persons (two parents of children: T. N and N. I, two researchers: MW and KS for medical science and welfare, respectively) classified and categorized the descriptions. When a description contained more than one element (e.g., painful sounds and difficulty hearing in a crowded room), it was aggregated in each category in duplicate. Differences in categorization among the four persons were discussed until they reached a satisfactory decision. We certified the sensory modality of each sensory issue by checking the content of the free-writing field. If there were obvious mistakes in choosing the sensory modality, we re-sorted them into a more appropriate sensory modality group (e.g., “confusions by a loud voice” in the “visual problem” choice). Typical descriptions of the categories are presented in Table 3.

**Table 3:** An example of the descriptions of sensory issues in a free-writing field.

A. Visual problems
Dazzling	外に出ると眩しい。蛍光灯やLEDの照明が特に眩しい 日中の運転中、道路が光を反射して眩しく気が遠くなりそうになる蛍光灯がチカチカして、学校の教室や図書館などで勉強するのが苦痛 昼は太陽の光が苦手です。 夜は対向車の上向きライトが苦痛です I get dazzled when I go outside. Fluorescent lights and LED lights feel especially bright to me. When driving during the day, the road reflects the sunlight and makes me feel faint I have a hard time studying in classrooms and libraries owing to the flickering of fluorescent lights During the day, I have a hard time with the sun’s rays. At night, the upward lights of oncoming cars are painful
Confusion of visual stimuli	視界に入ってきたもの全ての情報が頭に入ってくるため、物が多いところにいると辛い いろいろな物が目に飛び込んで来て刺激が大きすぎる。また、必要な情報に注目することができない 物が無造作にごちゃごちゃしている所を見ると頭の中で処理出来なくなる(圧迫感も作用している) It’s hard when I’m in a place where there are a lot of objects because all the information that comes in sight enters my mind Stimulation from all the things that come in front of our eyes is too strong. It also makes it difficult to pay attention to the information I need I cannot process the information in my mind when I see things jumbled up randomly (the feeling of pressure also plays a role)
Specific visual stimulus	赤色などのはっきりした色目、チェック柄を見た時 チカチカして見ていられない原色のコントラストの強いポスターなどの職場などの掲示物をみると不快になり、立て続けに見るといてもたってもいられずたまらなくなる 鳥よけで顔が書いてあるのがとにかく怖い I cannot stand the sight of bright colors such as vivid red and checkered patterns I feel uncomfortable when I see posters with strong contrasts of primary colors at work, and when I see them one after another, I cannot stand it any longer I’m scared of the faces on the posters used to keep birds away
Abnormal vision	全てにピントが合ってしまって奥域が認知できない 視界の周囲、視野の外側、焦点以外の部分がぼやけ、揺れ動いて見えるのが気になる 人の顔の判別が全くできないというほどではないが、なかなか人の顔が覚えられなかったり、長い付き合いの人でも全く違う人と間違えそうになったりする I feel that everything is in focus, and I cannot determine the depth I feel that the periphery or outside of my visual field, and areas outside the focus, appear blurred and shaky It’s not so much that I cannot distinguish people’s faces at all, but I have a hard time remembering people’s faces, or I almost mistake people I’ve known for a long time for completely different people
Difficulty in reading	字を読みづらい、長時間読めない 視覚的弁別(図形認識、活字の読み取り等)が苦手 Difficulty in reading letters or reading for long periods I experience difficulties in visual discrimination (figure recognition, reading print, etc)
B. Auditory problems
Specific sounds	子どもの泣き声、電子音、掃除機の音等が苦手 電子音が突き刺さる。レジのバーコードリーダーの音など 換気扇の音が苦手 キーボードをたたく音とか、物を咀嚼して食べる音、チャッて音が本当に無理です Not good with children’s crying, electronic sounds, vacuum cleaner sounds, etc Electronic sounds are painful for me, such as the beeps of a barcode reader at a cash register I have trouble with the sound of ventilation fans I really cannot stand the sound of keyboard tapping, or the sounds of chewing and eating
Selective listening	周りが静かな時には話がよく聞えますが、周りのうるさいと周りの音が聞こえて話があまり聞こえない 雑音の音量が下がらず、必要な音が聴き取れなくなることがある 常に人の声が複数あると聞きたい声の音に集中出来ないため、聞き取れない 雑音が聞こえると、指示が聞き取りにくい。 語尾や語頭が聞こえなくて、聞き直したり、間違ったことをする I can hear what someone is saying when the surroundings are quiet, but when the surroundings are noisy, I cannot hear much Sometimes the volume of the noise does not go down, and you cannot hear what you need to hear If there are multiple voices, I cannot focus on the voice I want to hear, so I cannot hear what someone is saying Difficulty hearing instructions when there is noise. I cannot hear the end or the beginning of a word, so I have to listen again or I misunderstand something
Many sounds	たとえばフードコートにいくと音が全部耳に入ってきてしまう 3つ以上の音声が全部同じ音量で聞こえて頭が混乱する 生活音全てが耳に入ってくる感じ。家電の音、交通網の音、自然界の音が一辺に聞こえて不穏になる For example, when I go to a food court, all the sounds (noises) come to my ears I hear three or more voices all at the same volume, and it messes with my head It’s like all the sounds of daily life enter my ears. The sounds of household appliances, traffic, and the sounds of nature are all around me, and it’s disturbing
Large sounds	大きな音や騒音がある環境下にいると疲れてしまう。具体的には頭が痛くなり辛い 自分と関係ない話でも、ある一定の大きさになると、頭が疲れてくる I get tired when I am in an environment with loud noises or noise. Specifically, I get a headache and have a hard time Even if it’s a story that has nothing to do with me, my head gets tired when it reaches a certain level of loudness
Sudden sounds	前もって想定される音ならば対処できるのですが、突然に発する音が苦手 学校の卒業式などに行われる「呼びかけ」は、突然 いろんなところから大声が聞こえるので苦しいです I can deal with sounds that are expected in advance, but I have a hard time with sounds that are sudden The “shouting” at school graduation ceremonies, for example, is distressing because you can suddenly hear loud noises coming from many places
C. Tactile problems
Clothes	服のタグは苦手。素材も綿はokだが、毛や化繊は苦手 肌触りが気に入らない服はかなりある。特に毛糸系 服のタグもそうですが、縫い目が肌に擦れる感覚がとても不快。緊張している時、ストレスがかかった時などは特に過敏になる I’m not good with tags on clothes. Cotton is OK, but not wool or synthetic fibers There are many clothes that I do not like the feel of, especially woolen ones Like the tags on clothes, the feeling of the seams rubbing against my skin is very uncomfortable. I am especially sensitive when I am nervous or under stress
Specific target	水に触れられない、洗濯や入浴が苦痛 小さい頃から粘土のような触感のものを触りたがらない I cannot touch water; thus, it is painful to wash and bathe I have been reluctant to touch things that feel like clay from a young age
Contact with humans	急に体を触られることが嫌い 痛いというほどではないが、くすぐったくて不快 手や腕にちょっとでも触られると気持ち悪い 家族でも嫌悪感を感じる I do not like to be touched suddenly. It’s not so much painful as it is ticklish and uncomfortable I feel uncomfortable if someone touches my hand or arm even a little. I feel disgusted even with family members
Itch	身体のところどころが痒くなる 外出したり考えたりして疲れた時が多い 自分の体の細かい凹凸が耐えられなくて、肌を掻きむしってしまう I get itchy in places on my body, mostly when I am tired owing to going out or thinking I cannot stand the fine unevenness of my body and I scratch my skin
D. Olfactory problems
Specific odors	芳香剤や柔軟剤の臭い、香水、コロンの臭い、タバコの臭いで頭痛になる。それに伴い思考力が低下して仕事や学習、読書といった事柄に影響が出る 車のガソリン臭が苦手で、乗ると臭いで酔ってしまう 些細な体臭でも気持ち悪くなる 自分の体臭も気持ち悪い Smell of air fresheners, fabric softeners, perfumes, cologne, and cigarettes give me headaches, which affects my ability to think, work, study, and read I cannot stand the smell of gasoline in the car, and when I get in the car, the smell makes me feel intoxicated Even the slightest body odor makes me feel sick. My own body odor also feels unpleasant
Various odors	あらゆる匂い(香水や芳香剤、柔軟剤等だけでなく、食べ物の匂いなども)がまとめて入ってきて、意識を失ってしまう 色んなものの臭いがして気持ち悪くなる All kinds of smells (perfumes, air fresheners, fabric softeners, food smells, etc.) come together and make me almost lose consciousness The smell of all kinds of things makes me feel sick
E. Taste problems	
Texture (touch)	キノコを食べると、噛んでも噛んでも形が残って、飲み込めなくて、気持ち悪い 鶏肉の皮の感覚、卵白の感覚がダメで卵かけごはんが食べられない When I chew mushrooms, the shape of it remains. So, I feel bad, and I cannot swallow them I cannot eat rice with eggs because of textures of chicken skin and egg whites
Specific foods	特に刺身などの生の海産物に代表される、一般の人が美味しいと思っているものが気持ち悪くて食べられない 給食で、ほとんど食べるものがない 白米くらい I feel bad and cannot eat what most people consider to be delicious (e.g., raw seafood such as sashimi) There is hardly anything that I can eat at school lunches. I can eat only white rice
Basic taste	化学調味料を多く含む食品(例:カップラーメン、スナック菓子等)や、塩味の強いものを食べると、頭痛・吐き気が起きる 甘すぎると食べたくなくなったり、気分が悪くなる(例:ミルクチョコレート、一部のデザートや清涼飲料水) Eating foods with a lot of chemical seasoning or a strong salty taste gives me headache and nausea (e.g., cup noodles, snacks) Too much sweetness makes me not want to eat or makes me feel sick (e.g., milk chocolate, some desserts, and soft drinks)
F. Proprioception problems
Body representations	左右がよくわからなくて、空間の中の自分の位置がわからない 道具の扱いが不器用。物にぶつかる、うまく避けられない I’m not sure which side is left or right, and I do not know where I am in space I am not good with tools. I keep bumping into objects and can hardly avoid it
Force controls	焦ってしまったり衝動性がつよいときはとくに、力加減ができない 自分ではそんなに力を入れていなくても、物が壊れる I cannot control my strength, especially when I’m impatient or impulsive Even if I do not put much effort into it, I sometimes break objects
Coordination/fine movements	手先が不器用だったり、体を頭で思ったように動かせないときがあること 針に糸が通せない I am clumsy with my hands or not able to move my body well in the way I think in my head at times I am not able to thread a needle
Posture controls	身体が真っ直ぐに保てない。すぐに動いてしまう 体幹が弱く、正しい姿勢の維持が困難 I cannot keep my body upright. I sometimes move easily My trunk is weak, making it difficult to maintain proper posture
G. Vestibular problems
Dizziness	調子が悪い時や心配事があると目眩やふらつき、気持ち悪さが出る スーパーなどの陳列棚を見たり交差点に行くと眩暈がする I get dizzy and lightheaded when I’m not feeling well or when I’m worried I get dizzy when I look at a display shelf in a supermarket or go to an intersection
Sense of balance	うまく歩けない。重心の取り方がいつまで経っても下手で、足首をぐねっと外側に思い切り捻る癖がついており、痛みはかんじないものの恥ずかしくてつらい。 エスカレーターでバランスを崩したり酔ったりする。ふと歩き方など体の動かし方が分からなくなって動けなくなる I cannot walk well. I’m not good at keeping my center of gravity, and I have a habit of twisting my ankle outward. Although I do not feel any pain, I feel embarrassed I lose my balance or get dizzy on escalators. I suddenly lose the ability to move my body, such as how to walk, and get stuck
Motion sickness	乗り物に乗ると疲れ果ててしまって通勤が困難 船酔い、ブランコ酔い I am exhausted from riding vehicles and find it difficult to commute Prone to seasickness and trapeze sickness
H. Others	
Temperature	急にもの凄く暑くて我慢できなくなり倒れそうな程しんどくなったり、一度寒くなったら熱源に当たらないと暖かくならないなど 暑いのか寒いのかが自分ではいまいち分からず(暑さより寒さのほうが感じにくい)、気温に合わない服装をして体調を崩すことがたびたびある I suddenly feel so hot that I cannot stand it and feel like I’m going to collapse. Once I feel cold, I have to turn a heater on to get warm I cannot tell if I’m hot or cold (it’s harder to feel cold than hot), and I often get sick by wearing clothes that do not match the temperature
Air pressure	気圧が下がるときに「あ、下がるな」というのがわかる。耳閉感や頭痛、気分が悪くなったり憂鬱になる 天候の変化により体調の良し悪しが変わる。気圧の変化に敏感 I can detect a drop in air pressure, “Oh, it’s going down.” It causes ear blockage, headache, feeling sick, or depression I feel better or worse depending on weather changes. I am sensitive to changes in air pressure
Multisensory	どれか一つが特別に強い訳ではなく、だいたいどの感覚において、その場面や状況によって予測なしに過敏になることがある 感覚全てが鋭い・強調されている感じ It’s not that any one sense is particularly strong, but that almost any sense can become unpredictably sensitive depending on the situation or circumstance All senses are sensitive and emphasized
Pain	四肢が慢性的に痛い、痺れる 怪我をしても気づかない。疲れていてもわからない Chronic pain or numbness in extremities I cannot notice when I am injured. I do not know if I am tired
Target temperature	温泉の温度、普通の人では平気に入っていける温度でもすごく熱く感じる レンジで温めた物も、ほかの人が普通に触れる程度でもとても熱く感じ、布巾などないと触れません As to temperature of the hot springs, even the temperature that normal people can enter without any problem, I feel it as being very hot As to microwaved foods, I feel so hot that I cannot even touch it without a cloth
Time	一旦集中すると体が疲労しきるまで何時間でも時間の経過を感じない。 もしくは行動するまでに時間がかかり、それらについても時間の経過を感じにくい Once I concentrate, I do not feel the passage of time for hours until my body is exhausted Or it takes a long time to act, and it is hard to feel the passage of time for that, as well

Examples of descriptions of sensory problems in each category are shown as original sentences in Japanese accompanied by English translations.

**Figure 1:**
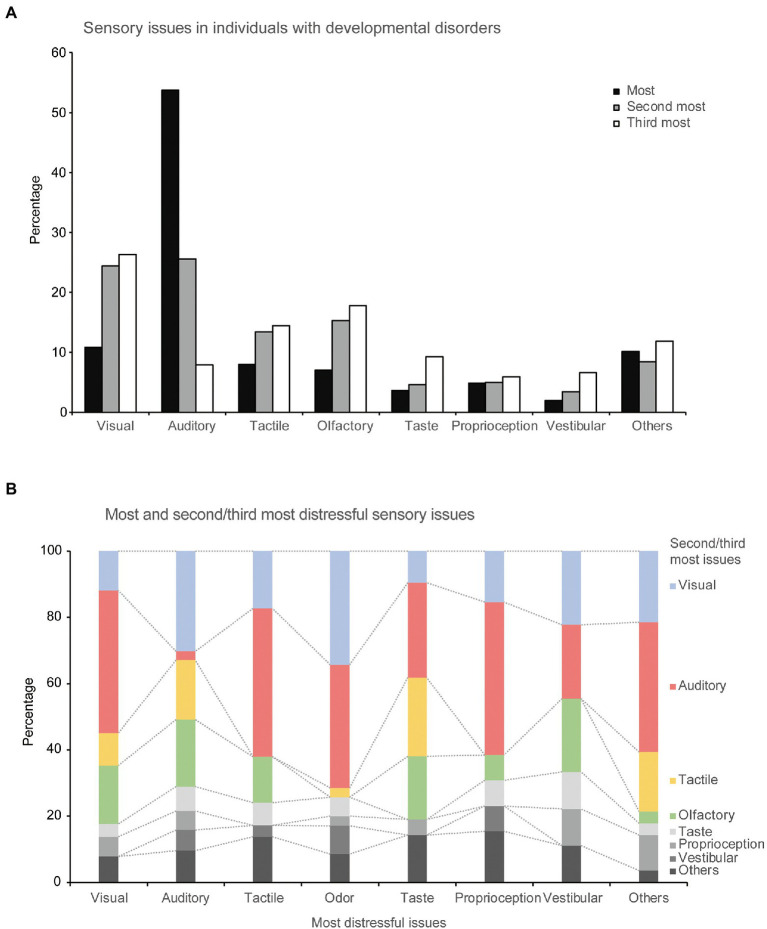
Sensory issues experienced by individuals with developmental disorders. **(A)** Percentage of each sensory modality in the most distressful, second-most distressful, and third-most distressful sensory issues, respectively. **(B)** Relationships between the most distressful and second/third-most distressful sensory issues.

## 3. Results

We investigated the frequency of each sensory modality in the most, the second-most, and the third-most distressful sensory issues categories.

### 3.1. Frequency of each sensory issue

Figure 1A shows the frequency of each sensory issue: the most, the second-most, and the third-most distressful issue. Approximately half of the most distressful sensory issues were auditory (223 out of 415 responses, 53.7%), followed by visual (45 responses, 10.8%) issues.

More than half the participants (262 of 415, 63%) mentioned and described the second-most distressing problem, in which auditory problems were still the most common (67 of 262 responses, 25.6%); however, visual problems became more prominent (64 responses, 24.4%), followed by olfactory (40 responses, 15.3%), and tactile (35 responses, 13.4%) problems.

More than one-third of participants (152, 36.6%) mentioned the third-most distressing problem. In this category, visual problems had the highest percentage (40 of 158 responses, 26.3%), followed by olfactory (27 responses, 17.8%), and tactile (22 responses, 14.5%) problems. Additionally, in the free-writing field about “other” sensory modalities, 10 participants reported severe sensory issues with multiple modalities. The relationships between the most distressful sensory issues and the second- and third-most distressing problems are shown in Figure 1B.

### 3.2. Differences in sensory issues among groups

**Figure 2:**
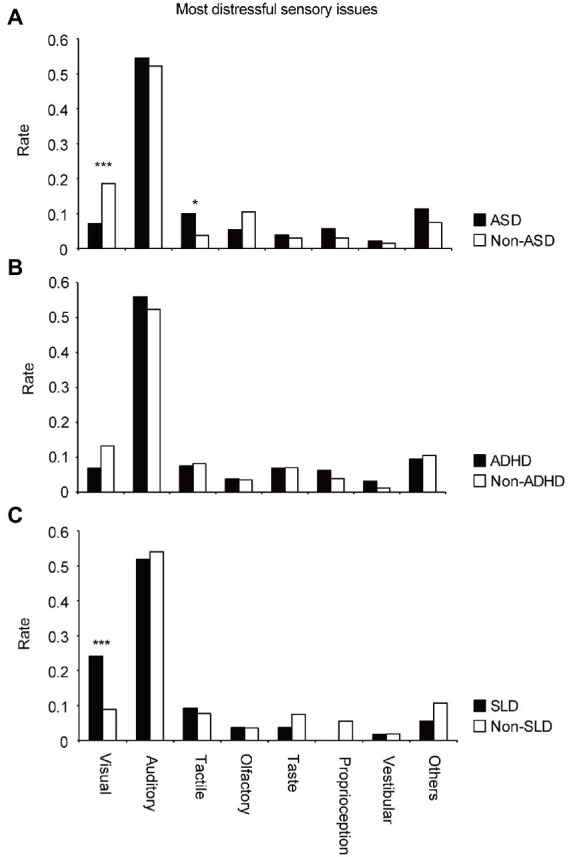
Differences in appearance rates of sensory issues in each modality for the most distressful sensory issues in the **(A)** ASD and non-ASD groups, **(B)** ADHD and non-ADHD groups, and **(C)** SLD and non-SLD groups.

To clarify the differences in sensory issues among the groups, participants were first divided into the ASD and non-ASD groups. For the most distressful sensory issue (Figure 2A), the *Χ^2^* test revealed significant group differences in the modalities of sensory issues (*Χ^2^* = 22.2, *df* = 7, *p* = 0.0023). Residual analysis (Supplementary Table S1A) showed that both groups had many auditory problems; however, the difference was not significant (adjusted residual = 0.42, *p* = 0.67). However, there were significantly more reports of visual problems (adjusted residual = 3.54, *p* = 0.00041; Supplementary Table S2A), after auditory problems in the non-ASD group. In the ASD group, there was a significantly higher number of tactile problems (adjusted residual =2.19, *p* = 0.028; Supplementary Table S2A) after auditory problems. For the second or third distressful problem (Supplementary Figures S1A,B), the *Χ^2^* test revealed no significant difference between the groups in the modalities of sensory issues (second-most distressful problem: *Χ^2^* = 9.67, *df* = 7, *p* = 0.21; third-most distressful problem: *Χ^2^* = 6.44, *df* = 7, *p* = 0.49). The results of the *Χ^2^* test and the residual analysis showed similar trends (most distressful problem: *Χ^2^* = 22.1, *df* = 7, *p* = 0.0024; second-most distressful problem: *Χ^2^* = 11.1, *df* = 7, *p* = 0.13; third-most distressful problem: *Χ^2^* = 5.83, *df* = 7, *p* = 0.56, Supplementary Table S3A), when the ASD-suspected cases (*n* = 11) were excluded.

Next, participants were divided into the ADHD and non-ADHD groups. For the most, second-most, and third-most distressful problem (Figure 2B; Supplementary Figures S1C,D), the *Χ^2^* test revealed that there were no significant differences between the two groups in the modalities of sensory issues (*Χ^2^* = 8.14, *df* = 7, *p* = 0.32; *Χ^2^* = 6.69, *df* = 7, *p* = 0.46; *Χ^2^* = 5.29, *df* = 7, *p* = 0.62, respectively). There was no difference in trend (*Χ^2^* = 7.32, *df* = 7, *p* = 0.40; *Χ^2^* = 6.57, *df* = 7, *p* = 0.48; *Χ^2^* = 5.19, *df* = 7, *p* = 0.64, respectively), when ADHD-suspected cases (*n* = 5) were excluded.

Third, participants were divided into the SLD and non-SLD groups. For the most distressful sensory issue (Figure 2C), the *Χ^2^* test revealed significant differences between the two groups in the modalities of sensory issues (*Χ^2^* = 17.0, *df* = 7, *p* = 0.017). Residual analysis (Supplementary Table S1B) revealed significantly more reports of visual problems (adjusted residual =3.66, *p* = 0.00025) following the auditory problems in the SLD group. For the second or third-most distressful problem (Supplementary Figures S1E,F), the *Χ^2^* test revealed no significant difference in the modalities of sensory issues (second-most distressful problem: *Χ^2^* = 1.99, *df* = 7, *p* = 0.96; third-most distressful problem: *Χ^2^* = 7.29, *df* = 7, *p* = 0.40). The results of the *Χ^2^* test and the residual analysis showed almost same trends (most distressful problem: *Χ^2^* = 15.9, *df* = 7, *p* = 0.026; second-most distressful problem: *Χ^2^* = 2.29, *df* = 7, *p* = 0.94; third-most distressful problem: *Χ^2^* = 7.33, *df* = 7, *p* = 0.40, Supplementary Table S3B), when the SLD-suspected cases (*n* = 2) were excluded.

### 3.3. Differences in sensory issues among age ranges

To clarify the differences in sensory problems among different age groups, participants were divided into four groups: minors (<18-years-old), adolescents (<30-years-old), middle-aged (<50-years-old), and late middle-aged (≧50-years-old). In this analysis, data for the most distressful, second-most distressful, and third-most distressful sensory problems were combined. The *Χ^2^* test revealed differences in the modalities of sensory problems based on participants’ age (*Χ^2^* = 41.3, *df* = 21, *p* = 0.0051). The residual analysis showed that there were significantly more reports of taste problems in minors (adjusted residual = 4.77, *p* = 0.000002, Supplementary Table S4) and fewer reports in the middle-aged group (adjusted residual = −2.58, *p* = 0.0099), compared with the other groups. Further, there were significantly more reports of visual problems in the middle-aged group (adjusted residual = 2.00, *p* = 0.046), compared with the other groups.

### 3.4. Free writing about the kinds of issues

**Figure 3:**
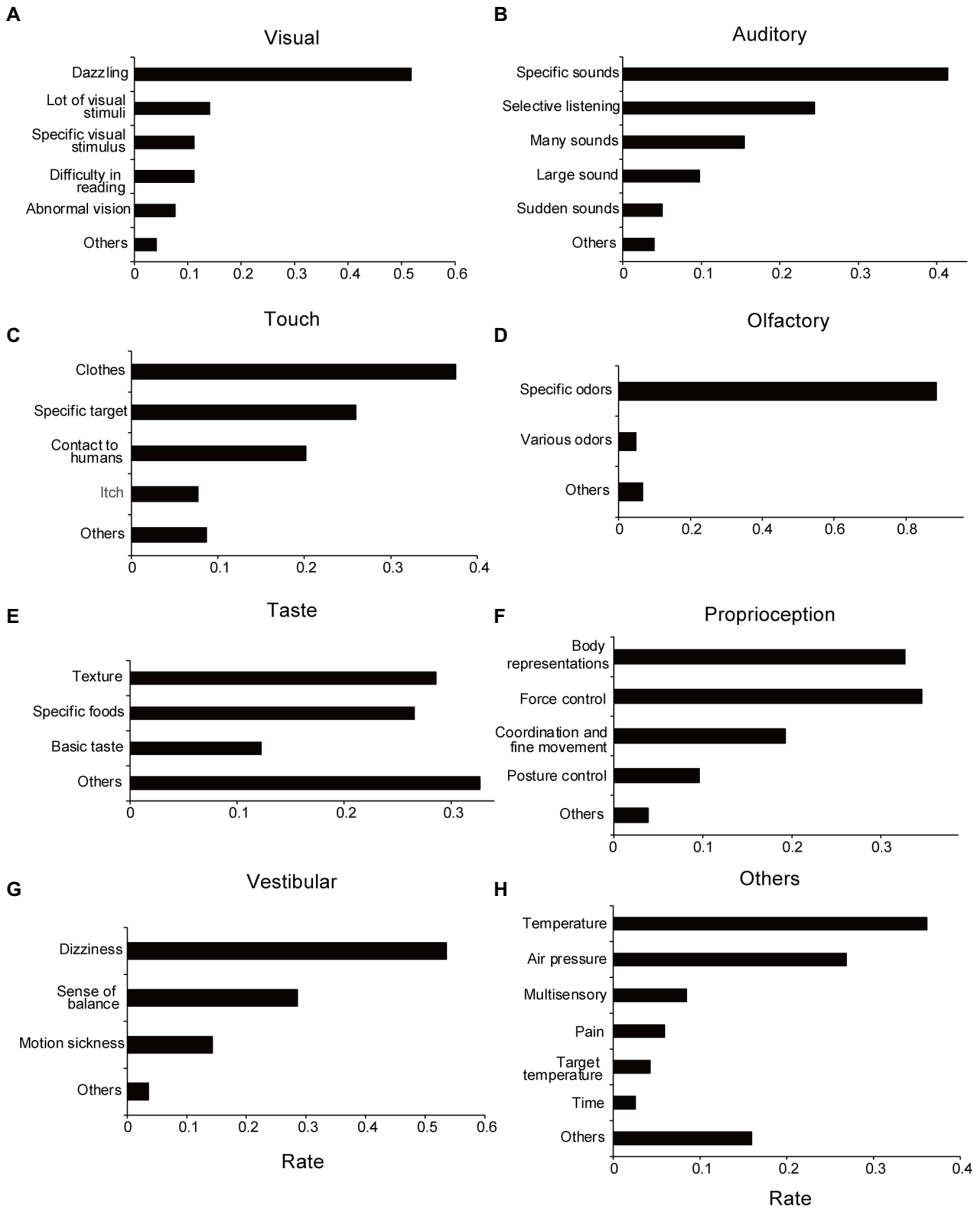
Categories of sensory issues extracted from free-writing fields. **(A)** Visual. **(B)** Auditory. **(C)** Tactile. **(D)** Olfactory. **(E)** Taste. **(F)** Proprioception. **(G)** Vestibular. **(H)** Others sensory issues. The rate of each category represents the ratio of response numbers to the total number of sensory issues aggregated by each modality. Note that when multiple categories are included in a single submission, they are counted in duplicate.

Regarding the free-writing field (“Please describe concrete examples of the issues. What are the triggers and reasons for their occurrence?”), the descriptions were classified and categorized, as described in the Methods section. The overall frequency of each problem categorized by sensory modality is shown in Figure 3, and examples of problems included in each category are shown in Table 2.

#### 3.4.1. Visual problems

Approximately half the participants reported that they disliked dazzling lights (Figure 3A; Table 2A). Responses related to detesting a specific visual target and confusion regarding multiple visual stimuli were also presented. These are related to visual hypersensitivity.

The *Χ^2^* test revealed marginally significant differences in visual problems (*Χ^2^* = 10.3, *df* = 5, *p* = 0.066; without suspected cases: *Χ^2^* = 9.32, *df* = 5, *p* = 0.097) between the ASD and non-ASD groups. Residual analysis showed that reading difficulty was frequently reported in the non-ASD group (adjusted residual = 3.07, *p* = 0.0021; without suspected cases: adjusted residual = 2.93, *p* = 0.0034; Supplementary Figure S2A; Supplementary Tables S5A, S6A). Moreover, between the ADHD and non-ADHD groups, the *Χ^2^* test showed marginally significant differences in visual problems (*Χ^2^* = 10.7, *df* = 5, *p* = 0.057; without suspected cases: *Χ^2^* = 10.3, *df* = 5, *p* = 0.068). Furthermore, residual analysis showed frequent reports of “dazzling” in the ADHD group (adjusted residual = 2.78, *p* = 0.0054; without suspected cases: adjusted residual = 2.94, *p* = 0.0034; Supplementary Figure S2B; Supplementary Tables S5B, S6B).

Next, the *Χ^2^* test revealed significant group differences in sensory issue modalities between the SLD and non-SLD groups (*Χ^2^* = 28.1, *df* = 5, *p* = 0.000035; without suspected cases: *Χ^2^* = 25.7, *df* = 5, *p* = 0.00010). Residual analysis revealed that “difficulty in reading” and “abnormal vision” were frequently reported in the SLD group (adjusted residual = 4.38, *p* = 0.00001 and adjusted residual =2.45, *p* = 0.014, respectively; without suspected cases: adjusted residual = 4.08, *p* = 0.000045 and adjusted residual = 2.52, *p* = 0.012, respectively; Supplementary Figure S2C; Supplementary Tables S5C, S6C). Contrastingly, there were significantly fewer reports of “dazzling” (adjusted residual = −2.53, *p* = 0.011; without suspected cases: adjusted residual = −2.40, *p* = 0.016; Supplementary Tables S5C, S6C) in the SLD group.

In summary, participants in the SLD group reported more reading problems and abnormal vision. Additionally, participants in the ADHD group may have had more problems with dazzling lights.

#### 3.4.2. Auditory problems

Most auditory problems were related to hypersensitivity, except for those classified as difficult-to-selective hearing, such as the inability to hear another person’s voice well in noisy conditions (Figure 3B). Of these, the most common answer was a description of specific sounds that were disliked, as well as loudness or unpredictable sudden changes in sounds. Examples of disliked sounds included children crying, electronic beeps, motor sounds, and human-generated sounds, such as those of chewing. Overall, participants tended to dislike sounds containing high-frequency components with a loud sound. Additionally, there were several descriptions in which various sounds, such as surrounding noises and speaking voices, were heard at the same volume, which made the participant feel tired. Regarding these auditory problems, there were no significant differences between the ASD and non-ASD groups (*Χ^2^* = 6.77, *df* = 5, *p* = 0.24; without suspected cases: *Χ^2^* = 6.40, *df* = 5, *p* = 0.27), the ADHD and non-ADHD groups (*Χ^2^* = 5.63, *df* = 5, *p* = 0.34; without suspected cases: *Χ^2^* = 4.88, *df* = 5, *p* = 0.43), and SLD and non-SLD groups (*Χ^2^* = 2.00, *df* = 5, *p* = 0.85; without suspected cases: *Χ^2^* = 2.86, *df* = 5, *p* = 0.72).

#### 3.4.3. Tactile problems

Most tactile problems were related to unpleasant tactile sensations (tactile hypersensitivity), such as problems with clothes, human contact, and specific targets (e.g., water; Figure 3C). Many participants described that they were concerned about clothes tags and disliked touching certain materials. Some participants described that they disliked human contact, such as handshakes, hugs, and contact in crowded trains. Some participants also stated that they disliked specific targets, such as water or paper-based materials, and could not touch them. Regarding tactile problems, no significant difference was found between the ASD and non-ASD groups (*Χ^2^* = 3.50, *df* = 4, *p* = 0.48; without suspected cases: *Χ^2^* = 3.45, *df* = 4, *p* = 0.49), the ADHD and non-ADHD groups (*Χ^2^* = 2.36, *df* = 4, *p* = 0.67), and the SLD and non-SLD groups (*Χ^2^* = 7.00, *df* = 4, *p* = 0.14). As for the ADHD and SLD groups, suspected cases were not included in the tactile problems. For the following sensory modalities, only the statistical results for all cases will be listed in the same way, if no suspected cases are included.

#### 3.4.4. Olfactory problems

Most responses were related to unpleasant odors. For example, odors related to humans, such as sweat; odors related to cars, such as gasoline; and daily life odors, such as kitchen waste, were typical responses (Figure 3D). Regarding olfactory problems, there were no significant differences between the ASD and non-ASD groups (*Χ^2^* = 0.72, *df* = 2, *p* = 0.70; without suspected cases: *Χ^2^* = 0.79, *df* = 2, *p* = 0.67), the ADHD and non-ADHD groups (*Χ^2^* = 4.21, *df* = 2, *p* = 0.12; without suspected cases: *Χ^2^* = 4.01, *df* = 2, *p* = 0.13), and SLD and non-SLD groups (*Χ^2^* = 2.44, *df* = 2, *p* = 0.29).

#### 3.4.5. Taste problems

There were many descriptions of the textures and certain foods (Figure 3E). There were few answers regarding “salty/sour/sweet/bitter/umami” (basic taste) tastes; however, some participants reported that they disliked strong tastes, such as strong seasonings (umami), salty, and sour tastes. Academically, texture is considered a somatosensory sensation, not a taste sensation. No significant differences were found between the ASD and non-ASD groups (*X^2^* = 0.082, *df* = 3, *p* = 0.99; without suspected cases: *Χ^2^* = 0.047, *df* = 3, *p* = 0.997), ADHD and non-ADHD groups (*Χ^2^* = 1.64, *df* = 3, *p* = 0.65), and SLD and non-SLD groups (*Χ^2^* = 1.89, *df* = 3, *p* = 0.60) regarding taste problems.

#### 3.4.6. Proprioception problems

Representative descriptions were categorized as “problems with body representations,” “problems with force controls,” “problems with coordination/fine movements,” and “problems with posture control” (Figure 3F). No significant differences were found between the ASD and non-ASD groups (*Χ^2^* = 3.24, *df* = 4, *p* = 0.52), ADHD and non-ADHD groups (*Χ^2^* = 5.09, *df* = 4, *p* = 0.28), and SLD and non-SLD groups (*Χ^2^* = 5.20, *df* = 3, *p* = 0.27; Without suspected cases: *Χ^2^* = 6.54, *df* = 4, *p* = 0.16).

#### 3.4.7. Vestibular problems

Vestibular problems were categorized as “dizziness,” “not good at balance,” and “prone to motion sickness” (Figure 3G). Regarding “prone to motion sickness,” only responses by the non-ASD group were classified in this category. Therefore, between the ASD and non-ASD groups, the *X*^2^ test revealed group differences in vestibular problems (*Χ^2^* = 11.4, *df* = 3, *p* = 0.0097; without suspected cases: *Χ^2^* = 10.7, *df* = 3, *p* = 0.013). The residual analysis showed fewer reports for “prone to motion sickness” in the ASD group (adjusted residual = −2.90, *p* = 0.0038; without suspected cases: adjusted residual = −2.83, *p* = 0.0047; Supplementary Figure S2D; Supplementary Tables S5D, S6D). Contrastingly, no significant difference was found between the ADHD and non-ADHD groups (*Χ^2^* = 2.44, *df* = 3, *p* = 0.49) and the SLD and non-SLD groups (*Χ^2^* = 1.59, *df* = 3, *p* = 0.66).

#### 3.4.8. Other problems

For “other sensory problems,” the descriptions were categorized as temperature-related problems, weather-related problems (such as temperature and atmospheric pressure), and pain-related issues (Figure 3H). Some participants described multiple sensory issues that could occur simultaneously, such as audiovisual, tactile, temperature, and olfactory problems. No significant difference was found between the ASD and non-ASD groups (*Χ^2^* = 9.09, *df* = 6, *p* = 0.17) or the SLD and non-SLD groups (*Χ^2^* = 3.37, *df* = 6, *p* = 0.76; without suspected cases: *Χ^2^* = 3.44, *df* = 6, *p* = 0.84). Contrastingly, the *Χ^2^* test revealed marginally significant differences between the ADHD and non-ADHD groups (*Χ^2^* = 12.3, *df* = 6, *p* = 0.055; without suspected cases: *Χ^2^* = 12.1, *df* = 6, *p* = 0.096). The residual analysis showed that reports regarding problems about “pain” were fewer in the ADHD group (adjusted residual = −2.36, *p* = 0.018; without suspected cases: adjusted residual = −2.32, *p* = 0.021; Supplementary Figure S2E; Supplementary Tables S5E, S6E). Additionally, as described in the Methods section, there were some descriptions of communication and interpersonal difficulties that could not be considered as sensory issues.

## 4. Discussion

This study investigated the characteristics of sensory issues in individuals with neurodevelopmental disorders using a web-based questionnaire and examined (1) the priority of the sensory issues; (2) the differences in sensory issues among individuals with ASD, ADHD, and SLD; and (3) the content of sensory issues with wide diversities.

Auditory problems accounted for nearly half of the most distressful sensory issues reported by participants, which coincides with previous studies suggesting the significance of auditory problems (23, 24). Therefore, the results suggest that auditory problems account for most distressful problems in individuals with neurodevelopmental disorders and that coping with auditory hypersensitivity and difficulty in selective listening are important for reducing issues in their daily life. Reduction of background noise by headphones or earplugs with a noise-canceling function is one of the most promising solutions to improve QoL for individuals with neurodevelopmental disorders (38).

As shown in Figure 1A, the frequency of visual problems was the highest after auditory problems. Particularly, in the SLD group, there was a high frequency of visual problems, (Figure 2C). In addition to typical visual hypersensitivity (e.g., aversion to dazzling lights), confusion about visual information and reading difficulties were also reported. Problems with difficulty in reading and abnormal vision, which were highlighted in the SLD group, may be related to the features of dyslexia. The SLD group may have included individuals with Irlen syndrome, which is also characterized by reading difficulties, abnormal vision, and visual hypersensitivity (19, 20, 39, 40). These characteristics were similar to those of the visual problems found in the SLD group. However, Irlen syndrome is not a medical diagnosis, and some researchers believe that some of its symptoms can be explained by eye movement disorders rather than sensory processing (21, 41, 42). Additionally, visual problems tended to be relatively more common in the middle-aged group (Supplementary Table S4). The effects of aging may be related to this result.

Contrastingly, in the ASD group, except for “other problems,” tactile problems were the most common, after auditory problems (Figure 2A). This result suggests that tactile problems are more likely to manifest in individuals with ASD. Most tactile problems were related to disturbing tactile sensations (e.g., clothes tags, clothing materials, and human contact), suggesting the existence of tactile hypersensitivity. Consistently, several previous studies have suggested that sensitivity for detection of tactile stimuli is high among individuals with ASD (43, 44), supporting the present result that aversion to touch is more critical than selective attention. The relationship between tactile temporal resolution and hypersensitivity in individuals with ASD (45) also supports this view. Additionally, among those who reported tactile problems, a certain percentage of participants also reported auditory problems (Figure 1B). Therefore, we must consider the possibility that tactile hypersensitivity prevents the use of headphones to cope with auditory hypersensitivity. Furthermore, various other sensory modalities were selected as distressful sensory issues (olfactory, taste, proprioception, vestibular, and others). Considering methods to cope with various sensory issues, this study clearly shows that we need to consider the co-occurrence of auditory and tactile problems.

In the free-writing analysis, we asked participants to describe the kinds of sensory issues that they face. Most responses were related to sensory hypersensitivity; in auditory, visual, and olfactory problems, there were cases where some distressful sensations such as specific stimuli, strong stimuli, and fluctuating stimuli were reported. In the case of auditory problems, examples of specific sounds included high-frequency sounds, such as children’s crying and electronic sounds. Loud and sudden sounds were also reported as distressful, suggesting that difficulty in stimulus prediction is related to hypersensitivity, as previously hypothesized (46). In other words, if a sensory stimulus is difficult to predict for the person, it will be perceived as a strong stimulus that arises suddenly. Regarding visual stimulus, it was noticeable that strong light such as sunlight was dazzling; however, the responses also suggested that visual stimulus at specific wavelengths, such as LEDs, primary colors, and blinking lights, are distressing. The fact that changing stimuli, such as blinking lights, is distressful, suggests a commonality with auditory problems. Regarding olfactory problems, descriptions of specific odors, such as daily life and car odors, were conspicuous. Most of the odors were generally so-called bad odors.

In other cases, various stimuli occurring simultaneously made respondents feel tired, even if individual stimuli were not distressful (confusions by “many sounds,” “lot of visual information,” and “various odors”). This type of hypersensitivity may be caused by sensory filtering for suppressing distractors or paying attention to targets (47, 48). We speculated that the difficulty in selective listening was caused by a similar mechanism; that is, it is difficult to suppress noise and voices other than that of the speaker’s (“the cocktail party effect”).

An additional analysis was conducted to examine changes in the priority of sensory issues by age groups. The result shows that the taste problem was relatively more common in the minor group, while it was less common in the middle-aged group. The taste problem may decrease with age. The fact that minors cannot choose the content of their meals, as represented by school lunches, may make the taste problem more apparent. Additionally, some taste problems (more than one-fourth) are related to food textures and should be strictly classified as somatosensory in the oral cavity. Considering this, it is appropriate to interpret “taste problems” in this survey as problems related to food (including taste, tactile, and olfactory), rather than simple taste problems. However, since there were some descriptions of basic tastes, we believe that there is indeed an issue with the taste itself. For proprioception and vestibular problems, a variety of answers were provided in the free-writing field. Since integration with other sensory signals is also important for body representation and postural control, and not only sensory information processing but also motor control issues are involved (49), the background needs to be carefully considered.

To implement evidence-based supports for the sensory issues in individuals with neurodevelopmental disorders, it is critical to clarify the neural basis of the sensory issues. Although deficits in sensory filtering, impaired predictions of sensory signals and so on (46, 48) have been proposed as possible mechanisms, the neural basis remains unclear. In this context, it is important to consider several syndromes with ASD characteristics, such as Fragile X syndrome, for which the causative gene (Fmr1) has been identified and the pathophysiology is becoming clearer. Moreover, Fragile X syndrome have prominent sensory issues such as auditory hypersensitivity, along with ASD characteristics (50–52). A variety of electrophysiological features have been reported, and it has been discussed that abnormalities in inhibitory PV neurons are associated with auditory hypersensitivity from studies of the model mice. It is also known in other mouse models (e.g., Shank3-KO mice) with a disturbance in the excitatory-inhibitory balance have characteristics related to the sensory hypersensitivity, such as enhanced tactile responses (53), and it is quite possible that a similar phenomenon may be responsible for sensory hypersensitivity in humans. Thus, basic research at the molecular level is promising for elucidating the mechanisms of sensory issues. Clarification of the basic mechanisms for each of the diverse sensory issues presented in this study will be essential for development of evidence-based support in the future.

This study had some limitations. First, diagnoses were based on participants’ self-reports, which included a small number of cases of “suspicion at the clinic.” Although the results showed almost same trends when the suspected cases were removed, these could be potential limitations. Additionally, regarding their diagnoses, participants were asked to respond based on their diagnosis at the time they were diagnosed. Thus, the ASD group includes participants with diagnosis of autism, Asperger’s syndrome, and pervasive developmental disorders. Moreover, many overlapping cases (e.g., ASD and ADHD) were included. The present analysis focused on the characteristics of each disorder; however, this is another limitation of this study, as overlap might cause unique sensory issues. Further investigation is needed with more cases with clear diagnoses. Second, the survey was restricted to individuals who were aware of their sensory issues and voluntarily participated in the survey. Therefore, it is possible that a sample bias exists when compared to the average representation of individuals with neurodevelopmental disorders. In addition to the possibility of differences in the manifestation of sensory issues, this might reflect differences in interest in the topic and hesitancy to participate in a research study. In addition, respondents’ cognitive capacity was not controlled for in this study because the respondents were widely recruited. Therefore, future surveys with diverse samples should control variables such as gender, social background, and cognitive capacity to reduce bias in responses. Third, the survey asked participants to list the sensory issues they experienced and assess their diversity and severity. Therefore, the degree of impact on QoL needs to be re-evaluated.

Among sensory issues, auditory problems (i.e., mainly hypersensitivity) are the most distressing in individuals with neurodevelopmental disorders. However, individuals with ASD also experience more tactile problems along with auditory problems. Contrastingly, individuals with SLD frequently report more visual problems. Among sensory issues, in addition to aversion to sudden, strong, or specific stimuli, they often reported confusions regarding multiple simultaneous stimuli. These results suggest that the diversity of sensory issues should be carefully considered when aiding individuals with neurodevelopmental disorders.

## Data availability statement

The raw data supporting the conclusions of this article will be made available by the authors, without undue reservation.

## Ethics statement

The studies involving human participants were reviewed and approved by Ethics Review Committee of the National Rehabilitation Center for Persons With Disabilities (29–175, 30–154, 31–109, 2021–136). Written informed consent for participation was not provided by the participants’ legal guardians/next of kin because: This is an anonymous web survey. An explanation of the purpose of the study was provided to the participants on the web, and after confirming the consent to participate in the study by ticking a check box (in case of minors, their guardians needed to agree on their behalf), the web survey was started.

## Author contributions

MW conceived the research and wrote the first draft. KH and KN conducted the web surveys. MW, KS, NI, and TN analyzed the data. All authors revised and approved the final manuscript.

## Funding

This study was partly supported by JSPS grants (JP 19H00532, JP 21H05053, JP19K22885, and JP22K18666) and MEXT grants (JP19H04921 and JP20H04595).

## Conflict of interest

The authors declare that the research was conducted in the absence of any commercial or financial relationships that could be construed as a potential conflict of interest.

## Publisher’s note

All claims expressed in this article are solely those of the authors and do not necessarily represent those of their affiliated organizations, or those of the publisher, the editors and the reviewers. Any product that may be evaluated in this article, or claim that may be made by its manufacturer, is not guaranteed or endorsed by the publisher.
